# *Ustilago maydis*: Dissecting the Molecular Interface between Pathogen and Plant

**DOI:** 10.1371/journal.ppat.1002955

**Published:** 2012-11-01

**Authors:** Armin Djamei, Regine Kahmann

**Affiliations:** Max Planck Institute for Terrestrial Microbiology, Marburg, Germany; Duke University Medical Center, United States of America

Fungal diseases of plants represent one of the most eminent threats to agriculture. Given the food needs of a growing world population and that more and more crops are devoted to fuel production, the necessity to develop crops with better resistance to disease is increasing. To accomplish this, the mechanisms that plant pathogenic fungi use to colonize plants need to be elucidated. As of now, there are only few examples/models in which this can be done on a functional, genome-wide level, taking into account both the pathogen and its host plant [1]. The fungus *Ustilago maydis (U. maydis)* is one of these examples. It is a member of the smut fungi: a large group of parasites infecting mostly grasses, including several important crop plants such as maize (Figure 1B), wheat, barley, and sugar cane. Smut fungi are biotrophs, i.e., parasites that need the living host plant to complete their sexual life cycle [2], [3]. They do not establish prominent feeding structures like the related, haustoria-forming rust fungi. During penetration, the host plasma membrane invaginates and completely encases the intracellular hyphae (Figure 1A), establishing an extended interaction zone [4] mediating the exchange of molecules between fungus and host. In contrast to most smut fungi that cause a systemic infection, remaining symptomless until the plant flowers, *U. maydis* can infect all above-ground parts of the maize plant but fails to spread systemically. *U. maydis* induces local tumors in which spores develop (Figure 1B) – a unique feature that allows detection of symptoms in corn seedlings less than a week after syringe infection with high levels of inoculum. This, together with the toolbox developed for reverse genetics, cell biology, and functional studies, has contributed to its status as a model for biotrophic basidiomycete fungi [5]. Here the current level of our understanding of the elaborate molecular crosstalk between *U. maydis* and its host plant will be discussed.

**Figure 1:**
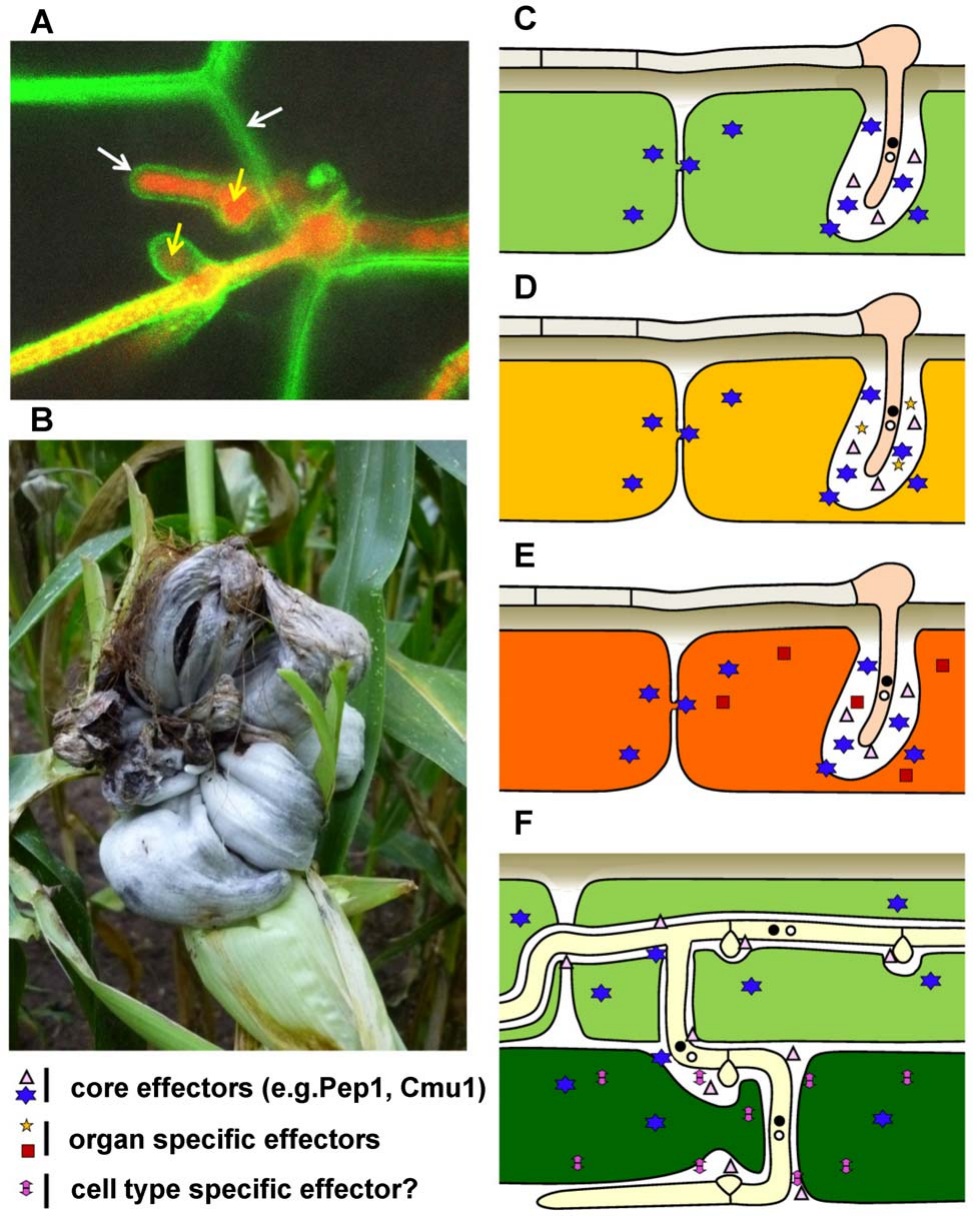
Disease symptoms and schematic presentation of effector cocktail use in different maize organs and tissues infected by *U. maydis*. A) Confocal microscopy of a *U. maydis* strain expressing cytosolic mRFP (yellow arrows) during intracellular growth in epidermal maize cells expressing PIN-YFP as a plasma membrane marker (white arrows). B) *U. maydis* tumor on field-grown maize plant (picture kindly provided by S. Krombach). C–F depict schematically the different tissues infected by *U. maydis* (the width of the interaction zone between hyphae and host plasma membrane is not drawn to scale): C) epidermal cell of an infected maize seedling (light green); D) epidermal cell of an infected mature leaf (yellow); E) epidermal cell of infected tassel (orange; F) epidermal cell (light green) and mesophyll cells (dark green) of infected seedling. Core effectors, organ-specific effectors, and cell type–specific effectors with either apoplastic or cytoplasmic function inside plant cells are indicated with different symbols.

## Secreted Effectors of *U. maydis*

Sequencing of the *U. maydis* genome and transcriptional profiling of different infection stages paved the path for the discovery of effector genes that govern interaction with the host plant. Effectors are broadly defined as microbe-derived secreted molecules that shape interaction with the host. In lower eukaryotes, effector identification is largely restricted to conventionally secreted proteins, as these can be recognized through the presence of a signal peptide. The *U. maydis* genome codes for about 550 predicted secreted proteins of which more than 50% are novel, lacking known Interpro domains [6], [7]. Many of these novel genes reside in gene clusters, are upregulated during host colonization, and encode effectors with a virulence function [6], [8]. Comparative approaches have shown that many of these novel effectors are also found in related smut fungi [8], [9]. They are either highly conserved or poorly conserved, leading to speculation that they may target equally conserved or variable host proteins, respectively [8], [9]. A recent study revealed that effector genes are differentially expressed in different infected maize organs and that some effector mutants affect tumor formation only in specific organs [10]. This mirrors the finding that different maize organs express distinct sets of proteins and suggests that the ability of *U. maydis* to induce tumors in different maize organs relies on its ability to reprogram different plant developmental states to suit its own needs, i.e., for fungal proliferation in tumor tissue [10]. Skibbe et al. [10] suggest a two-step process in which a first set of “core” effectors (Figure 1C–E) is used to suppress plant defense responses during penetration, and a second set of specific effectors responds to maize organ–specific properties (organ-specific effectors, Figure 1D, E). This indicates that *U. maydis* is able to sense the different developmental conditions of its host and reacts by secreting a cocktail of specifically tuned effectors for reprogramming these tissues. Linking effectors with virulence may thus require infecting different maize organs during different developmental stages rather than performing seedling infections with mutant strains, as is common practice nowadays. It is even conceivable that effectors could act in a cell type–specific manner, i.e., there could be effectors that are needed in epidermal tissue, and a different effector set might be required in mesophyll cells (Figure 1F). To identify such cell type–specific effectors, one could assume that the respective genes are transcriptionally upregulated in specific cell types and apply laser-dissection methods together with next-generation sequencing methods.

## Effectors Acting in the Biotrophic Interface between *U. maydis* Hyphae and Host

During the early phase of maize infection, *U. maydis* is recognized by the plant via conserved molecular patterns (PAMPs). This leads to salicylate-dependent defense responses, a typical response of plants to biotrophs [11], [12]. These plant defense responses are considered to be overcome/suppressed with the help of the set of core effector molecules that are upregulated during penetration. One of these core effectors is Pep1 (Figure 1C–F). *pep1* mutants are able to form normal appressoria but are arrested during penetration and induce strong plant defense responses that include the massive transcriptional upregulation of the maize-secreted peroxidase POX12 and an accumulation of H_2_O_2_ at attempted sites of penetration [13].

A biologically active Pep1-mCherry fusion protein localizes to the biotrophic interaction zone, suggesting that Pep1 is an apoplastic effector [13]. In a recent study, Pep1 is shown to interact directly with POX12 and to inhibit its activity [14]. Since the main sources for reactive oxygen species (ROS) in the apoplast are NADPH oxidases and peroxidases, Hemetsberger et al. [14] go on to show that biotrophic development of the *pep1* mutant can be partially restored by scavenging ROS as well as by silencing peroxidase POX12. The inhibition of plant peroxidases by Pep1 appears to be rather unspecific, as horse radish peroxidase is also inhibited [14]. Thus, the Pep1 effector targets an apoplastic peroxidase, a critical component of the PAMP-triggered defense program.

The recent analysis of a cluster of four genes transcriptionally upregulated during biotrophic development revealed that two of those genes, *pit1* and *pit2*, are important for tumor induction [15]. *pit1* as well as *pit2* mutants are able to colonize maize plants but are unable switch to strong proliferation and tumor induction. While *pit2* codes for a small secreted effector that accumulates in the biotrophic interphase, Pit1 is a transmembrane protein that localizes to the hyphal tips, moving early endosomes as well as vacuolar structures [15]. Intriguingly, *pit1* and *pit2* mutants induce transcriptional programs in infected maize plants that are indistinguishable, suggesting a yet unknown functional link between the apoplastic effector Pit2 and the transmembrane protein Pit1 [15].

## *U. maydis* Effectors Acting inside Plant Cells

In all plant-pathogen systems where respective analyses have been done, the repertoire of effectors consists of those functioning in the apoplast and those taken up by plant cells [16]. In *U. maydis*, a secreted chorismate mutase, Cmu1, serves as the first example of an effector that is translocated into the host cell. The *cmu1* gene of *U. maydis* is among the most strongly induced genes during plant colonization, and the Cmu1 protein is the most abundant fungal protein detected in the apoplast [17]. By complementation in yeast as well in vitro enzymatic assays, Cmu1 was shown to be a chorismate mutase. Chorismate is the branching metabolite of the shikimate pathway. Chorismate mutase catalyzes the conversion of chorismate to prephenate, which is further converted to an array of different phenylpropanoid compounds. Chorismate is also the precursor for aromatic amino acids and the plant defense hormone salicylic acid. In plants, the shikimate pathway resides in plastids [18]. Nevertheless, cytosolic plant chorismate mutases exist, and by two hybrid data, Cmu1 was shown to form heterodimers with the plastidic as well as the cytosolic isoforms [17], [19]. Immunolocalization studies indicate a cytosolic localization for Cmu1 after translocation into the host plant cell. In accordance with metabolic profiles of infected maize, a rechanneling of the chorismate flow is suggested by the cooperative action of the cytosolic maize chorismate mutase together with Cmu1, leading to a reduction of available chorismate for salicylic acid biosynthesis [17]. The ability of Cmu1 to spread locally to neighboring yet uninfected host cells (most likely via plasmodesmata) has been interpreted as metabolic priming, leading to lower salicylic acid levels, that prepares cells for the upcoming colonization by *U. maydis* [17]. Based on the presence of *cmu1*-related genes in other smut fungi [17], Cmu1 is also considered to be a core effector (Figure 1C–F).

## The Interface between *U. maydis* Hyphae and Host as the Site for Obtaining Nutrients

For the acquisition of nutrients, biotrophic fungi have to divert the metabolism of the host to provide them with nutrients via the extracellular, biotrophic interphase. This is mechanistically not trivial, as the extracellular release of monosaccharides has been shown to trigger plant defense responses [20]. *U. maydis* is shown to express a novel, plasma membrane-localized saccharose transporter (Srt1) during its biotrophic phase whose deletion strongly affects virulence. Srt1 is an H+-symporter specific for sucrose and displays an unusually high substrate affinity. These features are perfectly in-line with the needs of a biotroph: Srt1 guarantees efficient carbon supply and transports the disaccharide saccharose without producing apoplastic signals that trigger plant defenses [21]. Its functional role as a saccharose transporter during biotrophic development was corroborated by demonstrating that the *srt1* deletion phenotype can be complemented by the saccharose transporter AtSuc9 from *Arabidopsis thaliana*. Due to its low Km at a pH of 5.5 that is relevant for the apoplast, Srt1 can compete efficiently with plant saccharose transporters [22].

*U. maydis* infections lead to massive changes in the metabolome of infected plant tissue [11]. These metabolic changes could all result from repressing/modulating defense signaling pathways on different levels. Alternatively, the strongly elevated metabolites observed during infection could be of nutritional value for *U. maydis*, which would make this pathogen a “molecular farmer.” Yet another possibility would be that some of the metabolites induced are used by *U. maydis* to defend the habitat against other microbes. Compounds like DIMBOA (2,4-dihydroxy-7-methoxy-1,4-benzoxazin-3-one), sesquiterpenes, and defensins could conceivably have such a role. Genes involved in the synthesis of these compounds like *Bx1* (benzoxazinless 1), catalyzing the initial DIMBOA biosynthesis step, the sequiterpene cyclase *umi2*, and the defensin-related gene *umi11* are upregulated in tumor tissue [23].

With more genome sequences of related smut fungi with highly syntenic genomes becoming available [6], [8], [9], the identification of conventionally secreted effectors has become an easier task as many of these genes are only poorly conserved. Comparative genomics also allow for identification of effector sets that might have a role in determining host specificity. In addition, comparative genomics is a valuable tool for defining conserved effector domains for functional assays. However, to fully comprehend and appreciate the manipulative toolbox of a biotrophic pathogen like *U. maydis*, it will be necessary to decipher the functions of the >250 unknown effectors and to link these functions with the observed transcriptomic and metabolomic changes in the host. Given that many secreted effectors are species, genus, or family specific [9], [24]–[26], the huge challenge that needs to be met in the future is to find out whether eukaryotic plant pathogens target the same or different pathways in their respective hosts.
